# Yield and Predictors of Histologically Confirmed Colorectal Polyps in Adults Younger Than 45 Years Undergoing Non-screening Colonoscopy: A Retrospective Cohort Study

**DOI:** 10.7759/cureus.115892

**Published:** 2026-09-07

**Authors:** Godson Senyondo, Ahmed Shehadah, Ailsa K Lee, Warren H Megere, Imroz H Apon, Pranaya Baskaran, Jael M Nalukenge, AbdulSubhan Talpur, Sakhawat Usama, Minhaz Ahmad

**Affiliations:** 1 Gastroenterology and Hepatology, United Health Services (UHS) Wilson Medical Center, Johnson City, USA; 2 Internal Medicine, United Health Services (UHS) Wilson Medical Center, Johnson City, USA; 3 College of Medicine, Alfaisal University College of Medicine, Riyadh, SAU; 4 Internal Medicine, Gulu University, Gulu, UGA

**Keywords:** advanced colorectal neoplasia, colorectal adenoma, colorectal polyps, diagnostic colonoscopy, early-onset colorectal cancer, obesity, serrated lesions, young adults

## Abstract

Background

Early-onset colorectal cancer is increasing, but diagnostic colonoscopy yield below the routine screening age remains incompletely characterized. We evaluated histologically confirmed colorectal polyps in adults younger than 45 years undergoing non-screening colonoscopy.

Methods

This retrospective cohort study included 727 patients aged 18-44 years who underwent non-screening colonoscopy within a community health system during 2023-2024. Screening, surveillance, prior colorectal polyps or cancer, high-risk family history, inflammatory bowel disease, and hereditary colorectal cancer/polyposis syndromes were excluded. Outcomes required endoscopic identification and final histopathology. Logistic regression examined age, recorded sex, and body mass index (BMI), with aggregated indication and continuous-variable sensitivity analyses and a parsimonious neoplastic lesion model.

Results

Histologically confirmed polyps were present in 217/727 patients (29.8%; 95% confidence interval (CI), 26.6%-33.3%); yields were 17.4%, 33.0%, and 46.2% at ages 18-29, 30-39, and 40-43 years. Neoplastic lesions occurred in 139/727 (19.1%) and advanced colorectal neoplasia in 33/727 (4.5%), including four invasive cancers and one intramucosal carcinoma. In the primary model (n=724), adjusted odds ratios were 2.03 (95% CI, 1.35-3.06) for ages 30-39, 3.84 (95% CI, 2.31-6.38) for ages 40-43, and 1.85 (95% CI, 1.31- 2.62) for male sex. Obesity-class estimates were non-monotonic; class II was imprecise (1.84; 1.01-3.32), including after indication adjustment (1.79; 0.98-3.25). Primary model discrimination was modest (area under the curve, 0.692). The continuous age and the secondary neoplastic lesion model supported the age association; spline analysis suggested a non-linear association between BMI and polyp presence.

Conclusion

Histologically confirmed polyps and neoplastic lesions were not uncommon in this selected non-screening cohort. Findings support timely diagnostic evaluation when clinically indicated but do not establish population prevalence, justify screening before age 45, or validate a clinical prediction tool.

## Introduction

The incidence of early-onset colorectal cancer (CRC) has increased across several populations, shifting clinical attention toward colorectal neoplasia in adults younger than 50 years. Population-based studies document a rising disease burden in this age group, and national data also suggest increasing recognition of malignant colorectal polyps among younger adults [1-3].

Microsimulation modeling and subsequent recommendations from the American Cancer Society and the US Preventive Services Task Force lowered the starting age for average-risk CRC screening from 50 to 45 years [4-6]. These recommendations apply to asymptomatic, average-risk adults. They do not address the separate clinical question of lesion yield among adults younger than 45 years who undergo colonoscopy for symptoms, post-diverticulitis evaluation, or abnormal diagnostic findings.

Estimates from younger colonoscopy populations are heterogeneous because studies use different age ranges, referral indications, baseline-risk definitions, and outcome definitions. In particular, an endoscopic impression of a polyp may not be confirmed on histopathology. A clearly specified, histology-confirmed outcome is therefore important when describing the clinically verified polyp burden in this age group.

Our primary objective was to determine the patient-level yield and factors associated with histologically confirmed colorectal polyps in adults younger than 45 years who underwent non-screening colonoscopy. Secondary objectives were to characterize histology and quantify neoplastic lesions, advanced colorectal neoplasia, largest lesion size, intramucosal adenocarcinoma, and invasive CRC.

## Materials and methods

Study design and setting

This retrospective cohort study included adults aged 18-44 years who underwent non-screening colonoscopy at United Health Services (UHS) in upstate New York from January 1, 2023, through December 31, 2024. The study was designed to describe findings from non-screening colonoscopy rather than population screening.

Participants

Patients were identified using Epic SliceDicer (Epic Systems Corporation, Verona, Wisconsin, USA). One investigator reviewed referral and procedure records, pathology reports, medical and surgical histories, family-history fields, the problem list, and gastroenterology and primary care notes to establish eligibility and outcomes. Post-diverticulitis colonoscopy was classified as diagnostic evaluation to exclude underlying colorectal pathology, consistent with American College of Gastroenterology guidance [7]. Abnormal imaging indications included computed tomography findings of colonic wall thickening, mucosal irregularity, or a suspected lesion requiring endoscopic assessment. Each patient contributed once. Complete inclusion and exclusion criteria are organized by eligibility criterion in Table 1. 

**Table 1:** Study inclusion and exclusion criteria Personal and family histories were ascertained from the electronic record sources described above. CRC, colorectal cancer.

Criterion	Inclusion criteria	Exclusion criteria
Age and date	Age 18-44 years; colonoscopy from January 1, 2023, through December 31, 2024	Age outside 18-44 years or procedure outside the study interval
Procedure indication	Diagnostic or other non-screening indication, including post-diverticulitis follow-up	Average-risk screening, surveillance, or follow-up of a prior colorectal polyp
Prior colorectal disease	No prior colorectal polyp or CRC	History of colorectal polyps or CRC
Family history	No documented high-risk family history of CRC	At least one first-degree relative diagnosed with CRC before age 60 years, or at least two first-degree relatives diagnosed with CRC at any age
Inflammatory bowel disease	No known inflammatory bowel disease and no inflammatory bowel disease-directed evaluation	Known inflammatory bowel disease, colonoscopy performed because of suspected inflammatory bowel disease, or inflammatory bowel disease diagnosed by the data-abstraction date
Hereditary syndromes	No known hereditary CRC or polyposis syndrome	A hereditary CRC or polyposis syndrome in the patient, or such a syndrome in an immediate family member when the colonoscopy was performed because of that familial risk
Data availability	Sufficient clinical, endoscopy, and histopathology information for outcome adjudication	Missing core information required for eligibility or outcome adjudication

Data collection and outcomes

Variables included age, recorded sex, body mass index (BMI), primary indication, lesion size, and final histology. Age groups were 18-29, 30-39, and 40-44 years. BMI categories were underweight (<18.5 kg/m²), normal weight (18.5-24.9 kg/m²), overweight (25.0-29.9 kg/m²), and obesity classes I (30.0-34.9 kg/m²), II (35.0-39.9 kg/m²), and III (≥40.0 kg/m²). One primary indication was assigned per patient.

The primary outcome was an endoscopically identified colorectal polyp confirmed by final histopathology. Normal mucosa, non-polyp tissue, and suspected lesions without diagnostic polyp histology were not counted, as final pathology determined histologic classification, including serrated lesions. Patients with cancer qualified as polyp-positive only if a synchronous adenoma or an adenomatous component of the malignant lesion was documented.

Patients with multiple lesions were counted once. Pooled specimens established patient-level histology; individual size histology assignments required supporting specimen documentation. A neoplastic lesion comprised a conventional adenoma, a serrated lesion, an advanced neoplastic polyp, an intramucosal adenocarcinoma, or invasive CRC. Advanced colorectal neoplasia was defined as invasive CRC, intramucosal adenocarcinoma, high-grade dysplasia, villous/tubulovillous histology, an explicitly documented advanced neoplastic polyp, or a neoplastic lesion ≥10 mm. Intramucosal carcinoma was reported separately from invasive CRC.

Largest documented lesion size, including malignant lesions, was categorized as <10 mm, ≥10 mm, or indeterminate. Uncalibrated photographic estimates were excluded. A mass was defined descriptively as a lesion ≥3 cm in size, potentially with depression, ulceration, or necrosis.

Statistical analysis

Counts and percentages summarized categorical variables; Wilson 95% confidence intervals (CIs) were used to describe polyp yield. Multivariable logistic regression estimated adjusted odds ratios (aORs) with Wald 95% CIs for age group, sex, and BMI category. Reference groups were ages 18-29 years, female sex, and normal weight. Analyses used complete cases without imputation.

Sensitivity analyses adjusted for three indication groups: non-bleeding bowel symptoms (abdominal pain, diarrhea, bowel habits change, or constipation), rectal bleeding, and systemic/abnormal evaluation or follow-up (anemia, weight loss, abnormal imaging, post-diverticulitis evaluation, or other indications). A further model used continuous age (in five-year intervals), BMI (in 5-kg/m² intervals), sex, and indication. The same five-parameter specification was used for the secondary outcome of any neoplastic lesion.

Non-linearity was assessed using four-knot restricted cubic splines at the 5th, 35th, 65th, and 95th percentiles (age: 20, 30, 36, and 41 years; BMI: 19, 26, 32, and 45 kg/m²). For each predictor, two non-linear terms were tested against its linear specification using likelihood-ratio tests, while the other predictor remained linear.

Model performance was assessed using the area under the curve (AUC), Brier score, and Hosmer-Lemeshow test with approximately 10 groups, retaining tied predictions together. AUC 95% CIs were estimated using 5,000 bootstrap resamples with fixed fitted predictions. Performance was apparent and not optimism-corrected or externally validated.

Analyses were performed in Python 3.12 (Python Software Foundation, Fredericksburg, VA, US) using NumPy, pandas, and SciPy. Tests were two-sided; interpretation emphasized effect sizes and CIs. Sensitivity analyses were exploratory and did not undergo multiplicity adjustment.

Ethical oversight

The study was approved by the United Health Services (UHS) IRB; approval number: 15200. The requirement for informed consent was waived. Only de-identified analytic data were used for this study. All authors reviewed and verified the manuscript and take responsibility for its content.

## Results

The cohort included 727 patients aged 18-43 years; no patients aged 44 years were eligible. Regression analyses included 724 patients after excluding one with missing BMI and two with unclassifiable sex, retaining all 217 polyp-positive and 139 neoplastic outcomes. Histologically confirmed colorectal polyps were present in 217 patients (29.8%; 95% CI, 26.6%-33.3%). The proportion increased across age groups: 43 of 247 patients aged 18-29 years (17.4%), 119 of 361 aged 30-39 years (33.0%), and 55 of 119 aged 40-43 years (46.2%). Histologically confirmed polyps were identified in 102 of 267 male patients (38.2%) and 115 of 458 female patients (25.1%). The highest BMI-stratified proportions were observed in obesity class I (58/136, 42.6%) and obesity class III (37/90, 41.1%) (Table 2). 

**Table 2:** Patient characteristics and histologically confirmed polyps BMI was available for 726 patients; one value was missing. Percentages are calculated within each stratum. The two unclassified sex entries were retained in descriptive totals but excluded from regression. BMI, body mass index.

Characteristic	Total, n	Polyp, n	Proportion, %
Age group, years			
18-29	247	43	17.4
30-39	361	119	33
40-43	119	55	46.2
Recorded sex			
Female	458	115	25.1
Male	267	102	38.2
Unclassified	2	0	0
BMI category			
Underweight	23	5	21.7
Normal weight	188	35	18.6
Overweight	195	53	27.2
Obesity class I	136	58	42.6
Obesity class II	94	29	30.9
Obesity class III	90	37	41.1

Abdominal pain and rectal bleeding were the most frequent indications (210 patients each). Polyp yields by indication are shown in Table 3; estimates for smaller groups were descriptive. Aggregated indication groups comprised non-bleeding bowel symptoms (n=439), rectal bleeding (n=210), and systemic/abnormal evaluation or follow-up (n=78).

**Table 3:** Primary indication and histologically confirmed polyp yield Indication-specific values are descriptive. No multiplicity-adjusted comparisons were performed.

Primary indication	Total, n	Polyp, n	Yield, %
Abdominal pain	210	55	26.2
Rectal bleeding	210	68	32.4
Chronic diarrhea	168	46	27.4
Change in bowel habits	35	11	31.4
Chronic constipation	26	6	23.1
Anemia	25	13	52
Weight loss	21	8	38.1
Abnormal imaging	16	6	37.5
Post-diverticulitis follow-up	11	3	27.3
Other	5	1	20

Neoplastic lesions occurred in 139/727 patients (19.1%), and advanced colorectal neoplasia in 33/727 (4.5%). Four patients had invasive CRC (0.6%); one had intramucosal adenocarcinoma (0.1%). Histologic classification is shown in Figure 1.

**Figure 1:**
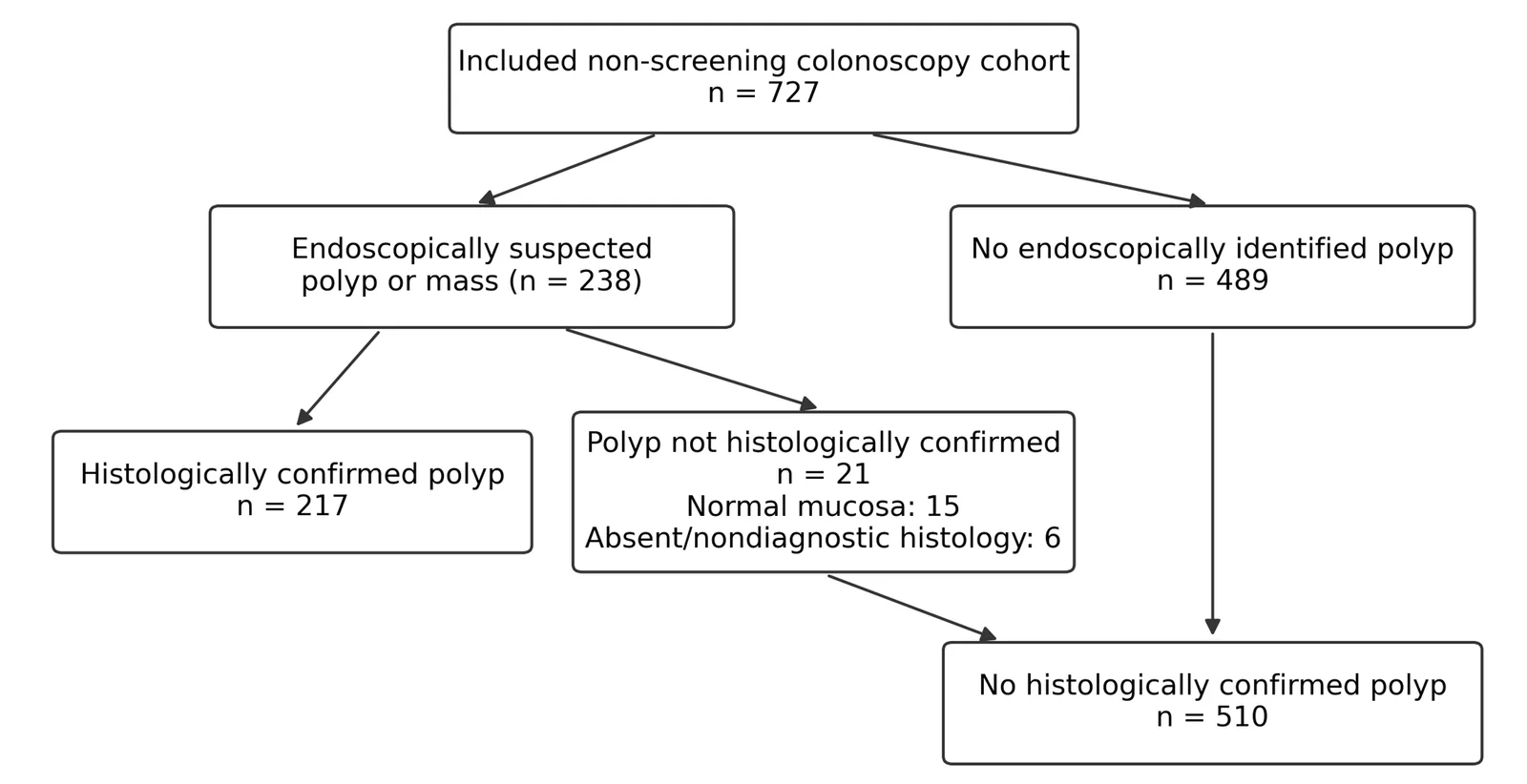
Histologic outcome classification within the included cohort

Among 217 polyp-positive patients, the largest documented lesion was <10 mm in 184 (84.8%), ≥10 mm in 31 (14.3%), and indeterminate in 2 (0.9%) (Table 4).

**Table 4:** Patient-level histology and clinically relevant outcomes Patients were classified once using the hierarchy: invasive CRC, intramucosal adenocarcinoma, advanced neoplastic polyp, conventional adenoma, serrated lesion, hyperplastic polyp, and inflammatory polyp. The advanced polyp category reflects an explicitly abstracted designation; the separate advanced neoplasia endpoint also applies size and histologic criteria and may overlap with other categories. Size is the largest documented dimension, including malignant lesions, and is not necessarily the diameter. CRC, colorectal cancer.

Outcome or category	n/denominator	%
Cohort-level outcome		
Any histologically confirmed colorectal polyp	217/727	29.8
Any neoplastic lesion	139/727	19.1
Advanced colorectal neoplasia	33/727	4.5
Mutually exclusive patient-level histologic category		
Conventional adenoma	76/727	10.5
Serrated lesion	35/727	4.8
Advanced neoplastic polyp (abstracted designation)	23/727	3.2
Hyperplastic polyp	71/727	9.8
Inflammatory polyp	7/727	1
Invasive colorectal cancer	4/727	0.6
Intramucosal adenocarcinoma	1/727	0.1
No histologically confirmed colorectal polyp	510/727	70.2
Largest documented colorectal lesion dimension		
<10 mm	184/217	84.8
≥10 mm	31/217	14.3
Indeterminate maximum	2/217	0.9

The primary complete-case model included 724 patients and 217 events. Relative to ages 18-29 years, adjusted odds were higher at ages 30-39 (aOR, 2.03; 95% CI, 1.35-3.06) and 40-43 (3.84; 2.31-6.38). Male sex was associated with higher odds (1.85; 1.31-2.62). Obesity-class estimates were 2.81 (1.67-4.72), 1.84 (1.01-3.32), and 3.00 (1.69-5.35) for classes I, II, and III, respectively. The class II estimate was relatively imprecise, and its CI approached the null (Table 5).

**Table 5:** Primary model and aggregated-indication sensitivity analysis Both models: n=724, 217 events. Reference groups: ages 18-29, female sex, normal weight BMI, and non-bleeding bowel symptoms. Indication-adjusted estimates are exploratory sensitivity results. aOR, adjusted odds ratio; CI, confidence interval.

Predictor	Primary aOR (95% CI)	p-value	Indication aOR (95% CI)	p-value
Age 30-39 vs 18-29 years	2.03 (1.35-3.06)	<0.001	2.00 (1.32-3.02)	<0.001
Age 40-43 vs 18-29 years	3.84 (2.31-6.38)	<0.001	3.75 (2.25-6.26)	<0.001
Male vs female	1.85 (1.31-2.62)	<0.001	1.84 (1.29-2.61)	<0.001
Underweight vs normal weight	1.48 (0.50-4.35)	0.481	1.36 (0.46-4.03)	0.579
Overweight vs normal weight	1.28 (0.78-2.12)	0.33	1.30 (0.79-2.15)	0.308
Obesity class I vs normal weight	2.81 (1.67-4.72)	<0.001	2.86 (1.70-4.83)	<0.001
Obesity class II vs normal weight	1.84 (1.01-3.32)	0.045	1.79 (0.98-3.25)	0.057
Obesity class III vs normal weight	3.00 (1.69-5.35)	<0.001	2.97 (1.66-5.31)	<0.001
Bleeding vs non-bleeding bowel symptoms	Not included	—	1.07 (0.73-1.58)	0.724
Systemic/abnormal evaluation or follow-up vs non-bleeding bowel symptoms	Not included	—	1.67 (0.98-2.84)	0.059

Adding aggregated indication produced similar estimates for age, sex, and obesity class I/III. The class II estimate was 1.79 (95% CI, 0.98-3.25; p=0.057). Indication estimates were 1.07 (0.73-1.58) for rectal bleeding and 1.67 (0.98-2.84) for systemic/abnormal evaluation or follow-up, compared with non-bleeding bowel symptoms. These CIs permit clinically relevant differences and should not be interpreted as establishing absence of indication-related effects.

In the continuous primary-outcome sensitivity model, the aOR per 5 years of age was 1.53 (95% CI, 1.33-1.77), and the modeled aOR per 5 kg/m² of BMI was 1.21 (1.09-1.34), adjusted for sex and indication (Table 6). Age spline terms did not materially improve fit (likelihood ratio χ²=0.56, 2 degrees of freedom (df), p=0.755). BMI spline terms improved fit (χ²=7.91, 2 df, p=0.019), suggesting departure from linearity. The linear BMI estimate therefore summarizes an imposed overall trend and is not evidence of a uniform dose-response relationship.

**Table 6:** Continuous-variable primary-outcome sensitivity and secondary neoplasia model Each model includes n=724; any-polyp events=217 and neoplastic-lesion events=139. Both models adjust simultaneously for all listed variables. Indication reference: non-bleeding bowel symptoms. The linear association between BMI and polyp presence is qualified by evidence of non-linearity (p=0.019).

Predictor	Any polyp aOR (95% CI)	p-value	Any neoplasia aOR (95% CI)	p-value
Age, per 5 years	1.53 (1.33-1.77)	<0.001	1.52 (1.28-1.80)	<0.001
Male vs female	1.82 (1.28-2.58)	<0.001	1.88 (1.26-2.80)	0.002
BMI, per 5 kg/m²	1.21 (1.09-1.34)	<0.001	1.22 (1.09-1.38)	<0.001
Bleeding vs non-bleeding bowel symptoms	1.12 (0.76-1.64)	0.57	1.52 (0.99-2.34)	0.055
Systemic/abnormal evaluation or follow-up vs non-bleeding bowel symptoms	1.55 (0.91-2.64)	0.107	1.34 (0.72-2.48)	0.351

The parsimonious neoplastic lesion model included 724 patients and 139 events. aORs were 1.52 (95% CI, 1.28-1.80) per 5 years of age, 1.88 (1.26-2.80) for male sex, and 1.22 (1.09-1.38) per 5 kg/m² of BMI. The rectal bleeding estimate was 1.52 (0.99-2.34), and the systemic/abnormal evaluation or follow-up estimate was 1.34 (0.72-2.48). Non-linear terms did not materially improve this secondary model for age (χ²=1.69, 2 df, p=0.430) or BMI (χ²=3.99, 2 df, p=0.136); limited precision remains relevant. The primary model had an apparent AUC of 0.692 (95% CI, 0.650-0.734), a Brier score of 0.189, and a Hosmer-Lemeshow χ²=5.76 (8 df; p=0.675). After indication adjustment, AUC was 0.697 (0.656-0.738). The secondary neoplastic lesion model had an AUC of 0.702 (0.653-0.748), a Brier score of 0.142, and a Hosmer-Lemeshow χ²=5.83 (8 df; p=0.667) (Table 7). The goodness-of-fit tests did not detect a gross lack of fit, but these in-sample assessments do not establish external calibration or clinical utility.

**Table 7:** Apparent discrimination and goodness of fit All models use 724 complete cases. AUC CIs use 5,000 resamples of outcomes and fixed predictions without model refitting or optimism correction. Brier scores depend on outcome frequency and should not be used alone to compare models with different outcomes. AUC, area under the curve; CI, confidence interval; df, degrees of freedom; HL, Hosmer-Lemeshow.

Model	AUC (95% CI)	Brier score	HL χ² (df)	HL p-value
Primary categorical	0.692 (0.650-0.734)	0.189	5.76 (8)	0.675
Indication-adjusted categorical	0.697 (0.656-0.738)	0.189	3.34 (8)	0.911
Continuous any polyp sensitivity	0.702 (0.661-0.742)	0.189	3.95 (8)	0.861
Parsimonious neoplastic lesion	0.702 (0.653-0.748)	0.142	5.83 (8)	0.667

## Discussion

In this selected cohort of adults younger than 45 years undergoing non-screening colonoscopy, 217/727 (29.8%) had a histologically confirmed colorectal polyp, 139/727 (19.1%) had a neoplastic lesion, and 33/727 (4.5%) had advanced colorectal neoplasia. Older age and male sex were associated with higher odds across primary and secondary analyses. Obesity estimates were generally elevated but non-monotonic, with uncertainty particularly evident for class II. These findings describe associations within a referred cohort and do not establish causal effects.

The distinction between an endoscopic impression and histologic confirmation materially affected the headline estimate. Endoscopy identified suspected polyps or masses in 238 patients; histology confirmed polyps in 217, while 21 lacked histologic confirmation. Using final pathology as the adjudication standard reduced the primary result from an endoscopic yield of 32.7% to a histologically confirmed yield of 29.8%. This definition prevents normal mucosa and other non-polyp tissue from being misclassified as polyps and aligns the main outcome with the biologic diagnoses used in the histologic analyses.

The age gradient is consistent with national data showing an increasing prevalence of lesions >9 mm across ages 40-59 and age-related differences in screening neoplasia [8,9]. Trivedi et al. analyzed 562,559 high-quality screening or diagnostic colonoscopies performed in individuals aged 18-54 [10]. Neoplasia yields were 26.6% at ages 40-44 and 32.1% at ages 45-49; advanced premalignant lesions occurred in 5.8% and 7.5%, and CRC in 0.53% and 0.58%, respectively. Age and male sex were associated with advanced colorectal neoplasia. Their registry included screening examinations and family history risk groups, whereas our cohort excluded screening and documented high-risk family history and included many younger adults.

El Hayek et al. studied 610 patients younger than 50 who already had adenomatous polyps; mean age was 42.9 years, 61% were male, and mean BMI was 27.5 kg/m² [11]. Of these, 299 were younger than 45. This male-predominant, overweight profile is compatible with our associations, but selection on an adenoma diagnosis prevents calculation of a directly comparable colonoscopy yield. Their 47.4% adenoma detection at follow-up among 156 returning patients was a surveillance finding, not baseline prevalence. Our denominator includes all eligible non-screening patients, including those without polyps; therefore, it is difficult to compare the 29.8% polyp yield and the 19.1% neoplastic lesion yield in our study with that follow-up percentage.

Shen et al. reported polypectomy in 2,050/10,250 adults aged 18-44 (20.0%) and analyzed 1,843 patients with 2,776 polyps after excluding incomplete reports [12]. Adenomas accounted for 47.6% of lesions, a lesion-level proportion rather than a patient-level neoplasia yield. Their cohort was 67.8% male, and polyp size and pedunculated morphology predicted advanced histology. The reported 5.3% advanced-histology frequency was from a different histologic definition and was within the selected polypectomy cohort; it is not directly comparable to our 4.5% advanced-neoplasia yield among all eligible colonoscopies. Our 29.8% yield also required histologic confirmation, whereas their 20.0% figure reflects the initial polypectomy denominator. Together, these studies show why referral selection, specimen confirmation, and patient-versus-lesion denominators must be explicit.

The continuous age analysis supported an age gradient beyond the selected category boundaries. In contrast, the categorical BMI estimates and significant non-linear terms for the primary outcome do not support a simple uniform dose-response relationship. The obesity class II primary estimate, 1.84 (95% CI, 1.01-3.32), was compatible with effects ranging from near-null to substantial; after indication adjustment, it was 1.79 (0.98-3.25). This small shift should not be presented as a qualitative reversal based on a p-value threshold. Subgroup size, exposure measurement, referral selection, and residual confounding may contribute. Prior work associates adiposity and metabolic/lifestyle factors with colorectal neoplasia or early-onset CRC, but populations and outcomes differ [13-16]. Gausman et al. found an association with male sex, while obesity prevalence was similar between early-onset CRC cases and controls [17].

Aggregating indications allowed adjustment for referral reason while retaining sparse clinical groups. The main age and sex estimates changed little, although residual confounding remains possible because a single primary indication cannot capture all symptoms and the third indication group was heterogeneous. Anemia, weight loss, and abnormal imaging findings remain descriptive due to small denominators. Post-diverticulitis examinations were retained as diagnostic follow-up rather than polyp surveillance, preserving the non-screening cohort definition.

The secondary neoplastic lesion analysis supports the relevance of age and sex associations beyond hyperplastic and inflammatory polyps. Nevertheless, apparent AUCs near 0.70 indicate only modest discrimination. Non-significant Hosmer-Lemeshow tests do not prove calibration, especially outside the development cohort. No risk threshold, screening strategy, or clinical prediction score was validated.

Clinically, these results reinforce that age below 45 years does not exclude important colorectal pathology. Increasing age within the under-45 population, male sex, and obesity may help contextualize pretest probability when a younger adult has an appropriate non-screening indication. The occurrence of invasive CRC and intramucosal adenocarcinoma also supports the avoidance of diagnostic delay when alarm features are present. However, this study does not establish a benefit for average-risk screening before age 45 years because it did not evaluate screening effectiveness, procedural harms, cost-effectiveness, or long-term outcomes.

Histology is a crucial step in CRC management. Fathi et al. discussed molecular imaging as a potential complement to anatomic imaging and tissue-based characterization for staging, response assessment, and personalized management of gastrointestinal malignancies [18]. This provides context for the downstream evaluation of invasive CRC. Our study did not evaluate molecular imaging, treatment effectiveness, or longitudinal oncologic outcomes and does not establish an indication for such imaging in patients with benign polyps.

Strengths include restriction to non-screening examinations, manual verification of eligibility, explicit definitions for high-risk family history and hereditary syndromes, histopathology-based outcome adjudication, and separate reporting of intramucosal adenocarcinoma and invasive CRC. Exact age strata were retained rather than using an open-ended 40-and-older category, which is important because the oldest observed patient was 43 years.

Limitations include the retrospective, single health-system design, referral selection, and limited generalizability beyond non-screening colonoscopy. Eligibility depended on available documentation. Smoking, race/ethnicity, metabolic comorbidities, medication exposures, procedural quality, lesion location, and endoscopist factors were unavailable or inconsistently recorded. Counts before and during eligibility exclusions were unavailable, preventing reconstruction of a complete selection flow.

Pooled specimens limited lesion-specific linkage of size, location, and histology. Narrative abstraction and two indeterminate maximum sizes introduced measurement uncertainty. Suspected lesions without diagnostic histology were unconfirmed rather than proven absent. Complete-case analysis may introduce bias, and no 44-year-old patient was represented. Small advanced-neoplasia and cancer counts precluded stable separate models. Sensitivity analyses were exploratory, and model performance was neither optimism-corrected nor externally validated. Prospective multicenter studies with standardized specimen handling and procedural quality measures are needed.

## Conclusions

Histologically confirmed colorectal polyps and clinically important neoplasia were not uncommon among adults younger than 45 years undergoing non-screening colonoscopy. The yield increased markedly with age within this young cohort, and male sex and obesity were independently associated with polyp detection.

These findings support timely, complete diagnostic evaluation when younger adults have appropriate clinical indications and emphasize the value of final histopathology for accurate outcome classification. They should not be interpreted as evidence for lowering the population screening age without prospective, multicenter trial confirmation and formal assessment of screening benefits and harms.
